# Provenancing Archaeological Wool Textiles from Medieval Northern Europe by Light Stable Isotope Analysis (δ^13^C, δ^15^N, δ^2^H)

**DOI:** 10.1371/journal.pone.0162330

**Published:** 2016-10-20

**Authors:** Isabella C. C. von Holstein, Penelope Walton Rogers, Oliver E. Craig, Kirsty E. H. Penkman, Jason Newton, Matthew J. Collins

**Affiliations:** 1 BioArCh, Departments of Archaeology & Chemistry, University of York, York, United Kingdom; 2 The Anglo-Saxon Laboratory, York, United Kingdom; 3 NERC Life Sciences Mass Spectrometry Facility, Scottish Universities Environmental Research Centre, East Kilbride, United Kingdom; Institut Català de Paleoecologia Humana i Evolució Social (IPHES), SPAIN

## Abstract

We investigate the origin of archaeological wool textiles preserved by anoxic waterlogging from seven medieval archaeological deposits in north-western Europe (c. 700–1600 AD), using geospatial patterning in carbon (δ^13^C), nitrogen (δ^15^N) and non-exchangeable hydrogen (δ^2^H) composition of modern and ancient sheep proteins. δ^13^C, δ^15^N and δ^2^H values from archaeological wool keratin (n = 83) and bone collagen (n = 59) from four sites were interpreted with reference to the composition of modern sheep wool from the same regions. The isotopic composition of wool and bone collagen samples clustered strongly by settlement; inter-regional relationships were largely parallel in modern and ancient samples, though landscape change was also significant. Degradation in archaeological wool samples, examined by elemental and amino acid composition, was greater in samples from Iceland (Reykholt) than in samples from north-east England (York, Newcastle) or northern Germany (Hessens). A nominal assignment approach was used to classify textiles into local/non-local at each site, based on maximal estimates of isotopic variability in modern sheep wool. Light element stable isotope analysis provided new insights into the origins of wool textiles, and demonstrates that isotopic provenancing of keratin preserved in anoxic waterlogged contexts is feasible. We also demonstrate the utility of δ^2^H analysis to understand the location of origin of archaeological protein samples.

## 1 Introduction

**Fig 1:**
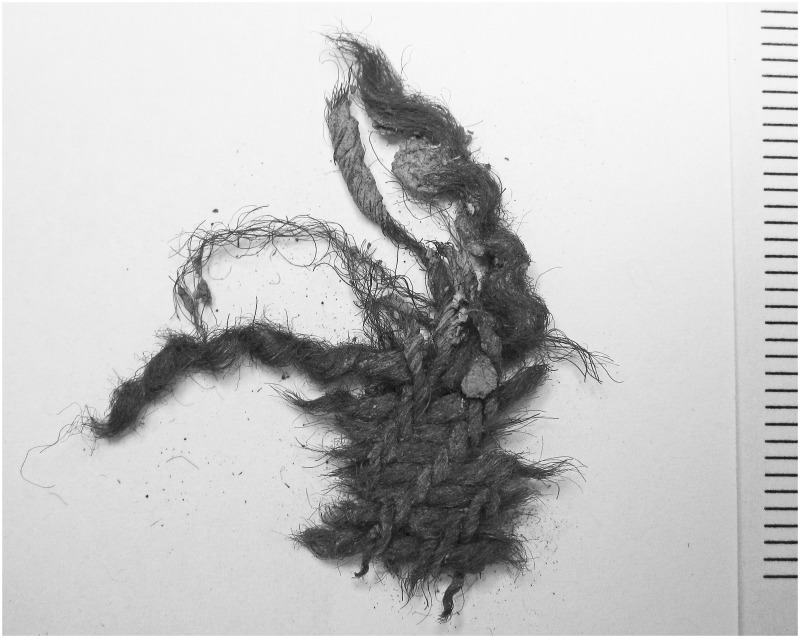
Fragment of sample 2897, a ZS 2/2 twill, the most abundant textile type at Reykholt/IS. It has been identified with *vaðmál*, a term used in Icelandic historical sources from the 11th century onwards for certain grades of cloth produced to regulated standards [11]. In this image, the warp runs vertically and weft horizontally. The warp yarn is more tightly spun than the weft, is spun clockwise (Z) where the weft is spun anti-clockwise (S), and contains a greater percentage of pigmented fibres. The weave structure is 2/2 twill: each yarn runs over-two-under-two yarns of the opposing system. Scale indicates mm.

Trans-European trade of raw wool and wool textiles was a cornerstone of economic and political development in the later Middle Ages (c. AD 1100–1500) [1,2]. The paucity of surviving historical documents from before 1100 AD makes it difficult to determine when and how these inter-regional exchanges developed, though the earliest such movements may date back to the 8th century AD [3]. Wool textiles are regularly found in anoxic waterlogged waste and latrine deposits in medieval rural and urban settlements in northern Europe [4–8] (Fig 1). Wool mostly consists of keratins (fibrous sulfur-rich proteins), which are widely analysed isotopically in forensic and ecological studies to establish the geographical origin of hair and feather samples [9,10]. Light stable isotope analysis of archaeological wool textiles is therefore a potential tool to interrogate the development of long-distance movements of these economically and socially significant objects. Use of these analyses must however take account of anthropogenic perturbation of geospatial isotopic signals in the tissues of domesticated herbivores, which have highly controlled diets. It must also consider the isotopic integrity of archaeological keratin samples preserved by anoxic waterlogging.

### 1.1 Understanding inter-regional movement of raw wool and wool textiles in northern Europe in the medieval period

Finds of medieval archaeological wool textiles from occupation sites are mostly small fragments. They are often parts of larger textiles which have been through several cycles of use and reuse, indicated by the presence of e.g. cut edges, sewing or deliberate folding, and damage due to wear. Unlike samples from graves, only a minority of fragments are identifiable to specific articles of clothing, furnishing or industrial textiles. Textile assemblages from settlements therefore represent the aggregate consumption of households or neighbourhoods. The relative frequencies of structural features of textiles (e.g. spin direction, thread count, weave type, dye use), all of which relate to the techniques of manufacture used to clean and align the fibres, produce the yarn and finally the cloth, braid or other object [12,13], and variation in wool fibre characteristics (e.g. diameter distribution, pigmentation) are compared within and between assemblages, and are used to classify textile finds into: (i) material typical of the region and therefore likely to be locally-made textiles in local styles, (ii) material which is not typical of the region and therefore potentially representing imports, and (iii) local copies of non-local goods. These identifications are rarely suggested on the basis of archaeological textile data alone, but within the context of data from contemporary sources, e.g. local finds of textile tools, iconography or documentary sources (see references above for examples).

A proportion of non-local textiles is expected in most assemblages, their number being especially marked in urban and high-status sites with access to exchange networks. Historical documents refer to inter-regional transfer of wool textiles: e.g. *pallia fresonica* [Frisian cloth] made in or traded through Frisia (coastal northern Germany and the Netherlands) in the 8th-10th century [14], *vaðmál* [standard cloth] from Iceland to mainland Europe from the 11th century (Fig 1) [11,15], *rays* and *stamforts* from England and Flanders to northern Italy, southern France and Spain from the 13th century [16], or *douayer* and *arras* cloths from northern France or the Netherlands to the Hanseatic cities in the Baltic from the same period [17]. Late medieval markets for standardised textiles were very large and production for them was often, though not exclusively, professionalised [1]. However historical documents almost never describe textiles in much technical detail, so that it is only rarely possible to link the contemporaneous term for the textile type to a specific archaeological textile type: three competing identifications have been made for *pallia fresonica*, for example [18,19,20], but there are no identifications of *douayer* or *stamforts*.

The historical sources of this information largely derive from mercantile activity and high-status consumption, and thus represent the activity and consumption of a smaller segment of the medieval population than archaeological textile fragments [21]. The presence of sheep and textile production equipment in the medieval archaeological record [22,23] indicates that domestic or small-workshop textile manufacture existed in parallel to specialist production, being especially prominent in the early medieval period and in late medieval rural districts [24,25]. Some non-professionally produced textile types were widely distributed: vaðmál is a historically attested example, and there are likely to have been many more. Wool textiles were also used in transport (sailcloth, sacking, tents [26]) and must also have travelled as personal possessions. Historical sources cannot therefore be taken as a summary of all wool textile movements of the period.

In summary, the extensive inter-regional movement of wool textiles described in medieval documents certainly underestimates the range of distribution and the types of textile moved in this period. Isotopic analysis of archaeological wool textiles to characterise the region of origin of their raw material is expected to identify additional flows of these artefacts, building on existing structural, fibre and dye analyses of these objects as typical or atypical of find site and period. This is especially significant for the period before the advent of substantial historical documentation of this economic sphere (c. AD 1200), and for portions of society or areas of Europe which are poorly recorded in historical sources.

### 1.2 The basis of isotopic provenancing of domesticated herbivore tissue

Systematic but complex patterns in the δ^13^C, δ^15^N, δ^2^H, oxygen (δ^18^O) and sulfur (δ^34^S) values of modern sheep muscle and wool proteins are present in Europe [27–31]. Thus, for example, Icelandic material is more depleted in ^15^N than all samples from elsewhere; samples from southern Europe show higher δ^13^C values compared to more northern regions; δ^34^S values are correlated with distance from the coast in Ireland. These patterns are caused by differences in the isotopic composition of graze plants, fodder plants and drinking water between different locations, which are reflected in the composition of consumer tissues. In northern Europe, where native terrestrial plants are entirely C_3_ [32], foliar tissue δ^13^C values are negatively correlated with both mean annual precipitation (MAP) and mean annual temperature (MAT), because the degree of discrimination against ^13^C in plant tissue during photosynthesis is strongly linked to plant responses to water availability [33]. Foliar tissue δ^15^N values are positively correlated with MAT and negatively correlated with MAP [34], probably largely indirectly, due to geographical dependence of plant mycorrhizal type and soil δ^15^N values [35]. Foliar water isotopic composition largely reflect local meteoric and groundwater inputs to plants [36], though significant and complex fractionation occurs in foliar water and solid tissues, in response to mechanisms of photosynthesis and water transport through the plant [37,38]. The isotopic composition of local meteoric water (and therefore that of foliar tissue) varies systematically with latitude and altitude, responding to the changing equilibria between evaporation and condensation in the water cycle [39]. In northern Europe (British Isles/Scandinavia/Baltic region), the correlations expected in foliar tissue between δ^13^C, δ^15^N or δ^2^H values and MAP or MAT were also observed in whole year samples of sheep wool, demonstrating the dominant influence of fresh graze plant composition on the isotopic composition of this tissue [31].

The geographical relationships described above can however be disrupted by farmers manipulating the isotopic composition of fresh pasture and fodders, thus providing animals with food and water which are isotopically inconsistent with local environment. Factors increasing δ^13^C values in herbivore tissues include grazing on marine plants [40], transhumance to higher altitude [41] and (theoretically) grazing in open pastures as opposed to under forest canopies, though this effect has not been directly demonstrated in modern domesticated herbivores [31,42,43]. Factors increasing δ^15^N values in herbivore tissues include grazing on haplophytic plants in salt marshes [44], transhumance to lower altitude [41], use of animal product or by-product fertilizers on pasture [45], (possibly) higher stocking level [46,47], higher diet protein content [48] and lower legume consumption [49]. Anthropogenic factors affecting herbivore tissue δ^2^H values are less well studied, but are likely to include the balance between fresh and dry fodders, and hence that between plant water and drinking water. Because animal and landscape management vary in response to local environment, economy and cultural factors, their isotopic effects on herbivore diet composition, and hence tissue composition, are uneven across regions [27] and through time [50]. Modern patterns of variability in herbivore tissue should not necessarily be expected to be identical to those in archaeological material.

Further complicating the comparison between modern and archaeological datasets are differences in the tissues typically sampled. Modern isotope work has focused on meat, milk and hair, because these tissues are of direct agricultural and industrial interest and/or can be non-invasively sampled [51]. In contrast, archaeological work has focused on bone collagen and tooth enamel, because they are most often preserved in archaeological deposits, e.g. [50,52]. Comparison of isotopic composition between tissues must take account not only of differences in composition, but also of the period of formation and turnover of the tissue in question [53]. Hair is not metabolically remodelled once formed [54], and its longitudinal isotopic composition thus reflects how that of ingesta change with time, in response to both graze plant annual composition cycles [38,55] but also season-specific farming practices such as stalling and foddering [29,56]. In contrast, the isotopic composition of herbivore bone collagen, which turns over continuously, integrates a longer period of dietary inputs compared to hair samples, and generates a more averaged diet signal [53]. For most skeletal elements this is likely to reflect diet over the whole lifetime of the individual animal.

Finally, comparisons between archaeological and modern samples must take account of changes in isotopic composition over longer time scales. Isotopic correlates of climate change have been identified in archaeological mammalian herbivore tissues [43,57]. In addition, fossil fuel burning has decreased δ^13^C values of modern organic tissue compared to preindustrial samples [58]. These effects, like those of farming and landscape management practices, are also likely to be regionally uneven, so that parallels between modern and ancient isotopic geospatial patterning must be interpreted with caution.

### 1.3 Isotopic integrity of archaeological keratin samples

Light stable isotopic analysis of archaeological keratinous tissues has so far been carried out only on material which is unusually well-preserved, for example under conditions of permafrost or desiccation [59,60]. In contrast, hair from anoxic waterlogged deposits, from which a high proportion of medieval European textiles derive, is clearly altered by diagenesis, either through chemical mechanisms (protein hydrolysis, deamidation, oxidation, breaking of S-S crosslinks) or microbiological activity (fungal and/or bacterial attack) [61–64]. These processes can add elements (O, H) to the fibre, remove elements (N, H) from the fibre, or cause protein chain scission, leading to loss of amino acids (AA). These processes can have isotopic effects [62,64]. The degree of degradation of hair fibres preserved by anoxic waterlogging must therefore first be assessed in order to understand the integrity of isotopic measurements of composition.

Establishing the degree of degradation of a hair fibre, and the effect of this degradation on isotopic and elemental parameters, is not straightforward [61]. Bulk fibre C:N atomic ratio (C:N_atom_) in particular has been used as an indicator of integrity [65,66], probably because of its ubiquity in assessments of bone collagen integrity [67], and because it is automatically generated during dual δ^13^C/δ^15^N IRMS analysis. However, in human, horse and sheep keratin fibre, there is experimental evidence of macroscopic alteration without significant alteration of δ^13^C or δ^15^N value or C:N_atom_, and in pigmented sheep wool, δ^13^C and δ^15^N value change has been detected without C:N_atom_ change [62,68]. In order to assess whether this measure is useful in wool samples preserved by anoxic waterlogging, C:N_atom_ data were compared with measures of degradation based on AA composition [62]. These variables reflect changes in the protein part of the fibre (i.e. ≥90% by mass) only, while C:N_atom_, also reflects the integrity of the non-protein moiety of the fibre.

**Fig 2:**
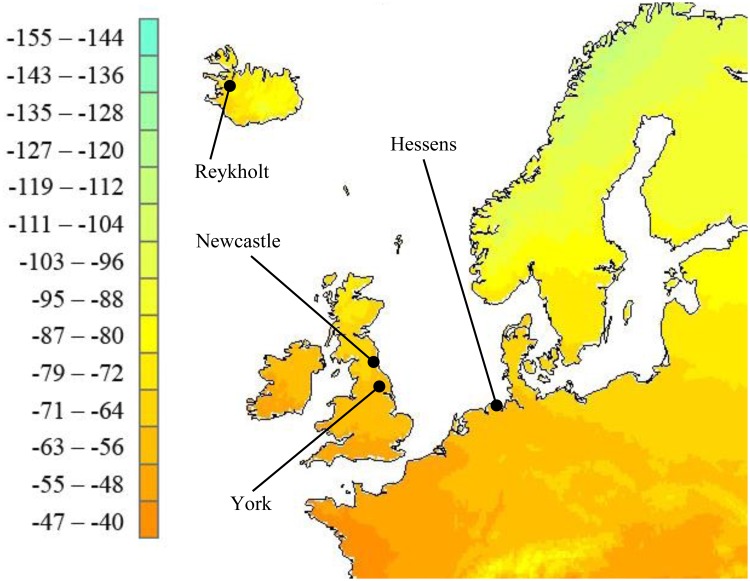
Map of annual mean δ^2^H values (in ‰) in precipitation in north-west Europe [73] with locations of archaeological assemblages tested.

**Fig 3:**
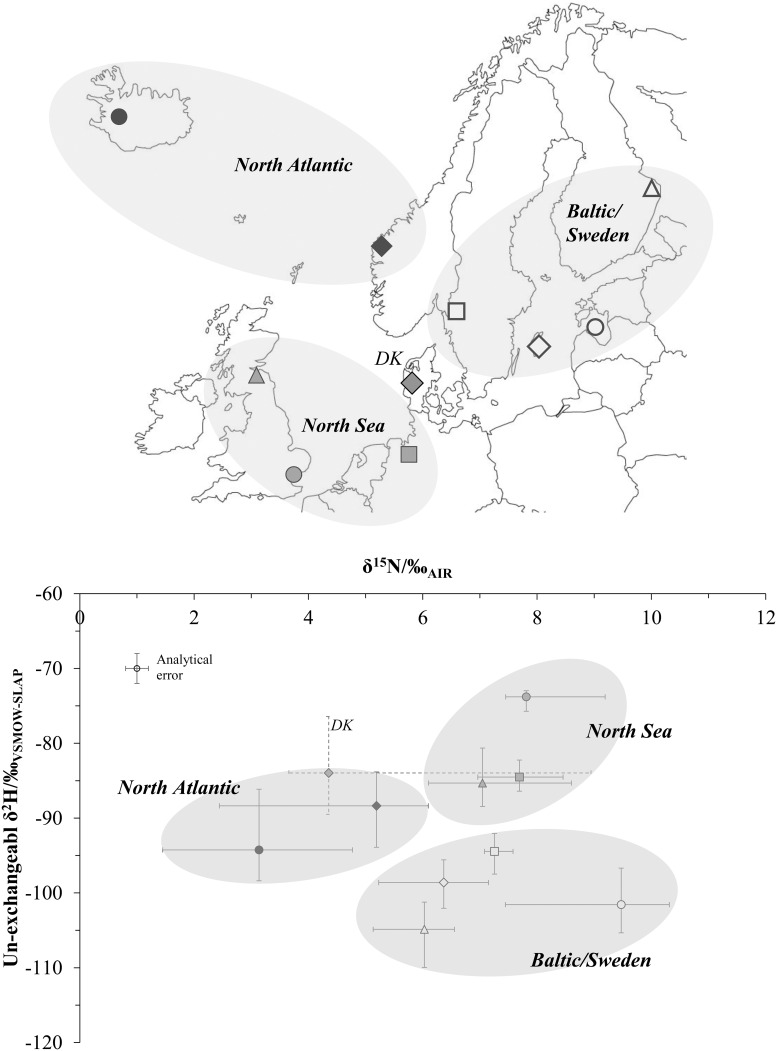
δ^15^N vs δ^2^H values for modern wool samples from northern Europe, redrawn from [31]. Each data point represents the median value (± maximum/minimum values) of wool samples (n = 9–10) from one flock. Flock locations are indicated in the insert map; point symbols and groupings match the main figure. DK indicates wool from Denmark.

In order to investigate whether the carbon (δ^13^C), nitrogen (δ^15^N) and non-exchangeable hydrogen (δ^2^H) isotopic composition of archaeological wool textiles could indicate their geographic origin, and thus the development of exchange patterns in this commodity, this study analysed wool samples from 7th-16th century AD contexts from Iceland (IS), north-east England (GB) and Frisia (coastal northern Germany [DE]) (Fig 2). All these regions have evidence of long-distance wool textile trades in the medieval period [1,3,15], and have productive natural and semi-natural C_3_ grasslands on a range of soil types, which were already present in the Middle Ages [69,70]. Existing δ^13^C and δ^15^N isotopic data from archaeological sheep bone collagen from these three regions shows good regional discrimination [50,71,72], as does modern wool δ^15^N and δ^2^H data (Fig 3) [31]. Results indicated that light stable isotopic analysis of this material was largely robust to diagenesis, and permitted the identification of non-local wool samples.

## 2 Material and Methods

This study compared sheep (*Ovis aries*) wool and bone collagen δ^13^C, δ^15^N and non-exchangeable δ^2^H values from 7 archaeological sites. The study tested the following hypotheses:

1. Authentic isotopic composition is preserved in medieval samples of wool keratin preserved by anoxic waterlogging.
2. Mechanical processing of wool during textile manufacture averages seasonal isotopic variation down the length of the fibre.
3. Modern and medieval geographic patterns of sheep protein isotopic composition are parallel.

### 2.1 Sample origin

The study analysed 83 textiles and 59 sheep bones from four occupation sites, both rural and urban, in Iceland, north-east England, and Frisia (Fig 2; Table 1; S1 Table).

**Table 1:** Archaeological find sites of samples tested in this study.

Site	Country	Code	Location	Site type	Latitude/ longitude	Altitude/m	Period selected	N textile samples analysed by IRMS (by HPLC)	N bone samples	Reference
Reykholt, Borgarfjörður	IS	RKH	Reykholt	Rural, inland	64.665/-21.292	45	C11-16	21 (21)	7	[6]
Hessens, Kreis Wilhelmshaven	DE	HSS	Hessens	Rural, coastal (salt marsh)	53.518/8.070	0	C7-8	10 (10)	7	[4,5,74]
16–22 Coppergate, York	GB	YCG	York	Urban, inland	53.958/-1.081	20	C9-15	21 (21)	16	[7,12,75]
6–8 Pavement (Lloyds Bank site), York	GB	YLB	York	Urban, inland	53.958/-1.080	21	C11	11 (12)	0	[76]
Rear of 7–15 Spurriergate, York	GB	YSG	York	Urban, inland	53.958/-1.083	18	C11	4 (5)	0	[77]
Black Gate, Newcastle upon Tyne	GB	NBG	Newcastle	Urban, inland	54.969/-1.611	19	C15-16	12 (12)	14	[78]
Queen Street, Quayside, Newcastle upon Tyne	GB	NQS	Newcastle	Urban, inland	54.969/-1.607	12	C13	4 (4)	15	[79]

Specimen numbers for all samples are given in S1 Table. All necessary permits were obtained for the study, which complied with all relevant regulations. Permission to sample assemblages was given by Guðrún Sveinbjarnardóttir of Þjóðminjasafn Íslands, Reykjavík (RKH); Christine McDonnell at York Archaeological Trust (YCG, YLB); Andrew Parkin at the Great North Museum, Newcastle (NBG, NQS); Klaus Tidow, former director of the Neumünster Textilmuseum (HSS textiles); and Annette Siegmüller, Niedersächsisches Institut für historische Küstenforschung, Wilhelmshaven (HSS bone).

Samples from Reykholt are deposited with the Þjóðminjasafn Íslands, Reykjavík. Samples from York (YCG, YLB) are deposited with the York Archaeological Trust, York; textiles from YSG are on long-term loan to The Anglo-Saxon Laboratory from MAP Archaeological Consultancy Ltd. Samples from Newcastle (NBG, NQS) are deposited with the Great North Museum, Newcastle upon Tyne, but NBG textiles are on long-term loan to The Anglo-Saxon Laboratory. Textiles from Hessens were deposited with Textilmuseum Neumünster but samples are on long-term loan to The Anglo-Saxon Laboratory, while the bone has been deposited with the Niedersächsisches Institut für historische Küstenforschung, Wilhelmshaven.

### 2.2 Sample types

Wool samples included both unprocessed raw staples (the clusters of wool fibres into which the fleece naturally grows) and completed textiles (combed, spun and woven). In intact staples, the fibres lie in parallel, with the segments grown in each season level with each other; the fibres must all come from the same animal; and each fibre represents total growth between shearing dates, typically at least annually [53,80]. In finished textile products, combing or carding the fibres in preparation for spinning de-aligns sections grown at the same time. A sample of >50 fibres from a yarn therefore derives from all parts of the year, and also probably several staples, though probably not more than one animal, given the quantity of material which can be processed at a time with medieval hand tools [24,81]. No difference in processing effects was expected between textile construction types (e.g. tabby, twill, knit).

Textiles were dated by context to the 7th–16th centuries AD. Bone samples were selected from the same or contemporaneous contexts.

### 2.3 Sample preparation

A fragment of textile, weighing approximately 0.1 g, was selected from each find. Dirt and inherent lipid were removed by sonicating in ultra-pure water (ELGA Purelab Ultra, Marlow, UK; 2 x 30 mins), and four times in mixtures of dichloromethane and methanol (both HPLC grade, Fisher Scientific, Loughborough, UK; 2 x 30 mins in 2:1 v/v mixture; 2 x 30 mins in 1:2 v/v mixture).

In order to examine the degree of seasonal variability in a single raw sample, sample 2950 was subdivided by cutting across the lock into ten segments c.1cm in length, representing sequential periods of growth, before being washed as described above.

In order to examine the isotopic effects of washing procedures which may have been employed during conservation or laboratory workup of hair or wool samples, sample 4120 was washed using a range of these methods, as follows: (1) Triton X100 (Fisher Scientific, Loughborough, UK) [76]; (2) sodium dodecyl sulfate (Melford Laboratories Ltd, Ipswich, UK) [82]; (3) 2% solution disodium EDTA (Sigma-Aldrich, St Louis, MO, USA) [7]; (4) pyridine (Fisher Scientific) [83]; (5) dichloromethane/methanol (both HPLC grade, Fisher Scientific) [84]; (6) deionised water, (ELGA Purelab Ultra, Marlow, UK) [85]; (7) 2:1 chloroform (VWR International, Fontenay-sous-Bois, France) /methanol (as above) [86]; (8) 2:1 methanol/chloroform (both as above) [87]; or (9) no treatment [9,59].

For collagen extraction, 0.5–1.0 g of bone chunks was demineralised in 0.6 M HCl (aq) at 4°C. Samples were rinsed with distilled water, then gelatinised in pH 3 HCl (aq) at 75°C for 48 h. The supernatant containing the collagen was filtered (30 kDa, Amicon^®^ Ultra-4 centrifugal filter units, Millipore, Billerica, MA, USA), frozen, and lyophilised.

### 2.4 Isotopic analyses

In weighing cleaned samples for isotope analysis, whole fibres were selected from staples; for finished textiles, cross-sectional samples of a single yarn (typically >50 fibres) were taken.

δ^13^C and δ^15^N isotope value analyses (except bone collagen samples from YCG) was carried out at the Natural Environment Research Council Life Sciences Mass Spectrometry Facility (NERC LSMSF) in East Kilbride. Aliquots (0.7 mg) of both bone and keratin were weighed into 4 x 3.2 mm Sn capsules (Elemental Microanalysis, Okehampton, UK). δ^13^C and δ^15^N isotope ratio mass spectrometric (IRMS) analyses were carried out on a ThermoElectron Delta Plus XP (Thermo Fisher Scientific, Bremen, Germany) with Costech ECS 4010 elemental analyser (Costech International, Milan, Italy); internal standards were a gelatine standard, two alanine single AA standards enriched with ^13^C and ^15^N respectively, and a ^15^N-enriched glycine single AA standard. C and N content and C:N_atom_ ratios were calculated using a tryptophan standard.

Bone collagen from YCG was analysed at the Stable Isotope Laboratory in the School of Archaeological, Geographic and Environmental Sciences, University of Bradford. Duplicate aliquots of 1.0 mg were weighed in 4 x 3.2 mm Sn capsules. Their isotopic composition was measured using a Finnigan Delta Plus XL isotope ratio mass spectrometer, coupled with a Thermo Flash EA 1112 elemental analyser via a Finnigan Conflo III interface (all Thermo Fisher Scientific). The instrument was calibrated using both laboratory (Fish gel, BLS) and international standards (IAEA 600, N1 and ANU sucrose).

All δ^2^H composition analyses were carried out at NERC LSMSF. 0.1 mg washed wool was weighed into 4 x 3.2 mm Ag capsules (Elemental Microanalysis, Okehampton, UK and Pelican Scientific, Stockport, UK). δ^2^H values were measured with a Thermo Fisher Scientific Delta V Plus with a TC/EA high temperature furnace. The contribution of exchangeable hydrogen was calculated using keratin standards BWB-II (bowhead whale baleen), CFS (chicken feathers), ISB (Icelandic black-legged kittiwake, Rissa tridactyla, feathers) and WG (Willow grouse, Lagopus lagopus, feathers) [10,88] and a comparative equilibration method [89]. The δ^2^H values of the non-exchangeable H in the four keratin standards was previously determined using a steam equilibration technique [90]. Calculation of non-exchangeable δ^2^H composition assumed a fractionation factor of α = 1.080 (ε_x-w_ = 80 ‰).

δ^13^C, δ^15^N and δ^2^H values are reported in per mille (‰) relative to VPDB, AIR and VSMOW respectively. Analytical error was better than 0.25‰ in δ^13^C and 0.35‰ in δ^15^N measurements (both 1σ). Analytical error for δ^2^H isotope measurements differed between substrates [53]: it was better than 4‰ in keratin, and within 9‰ in collagen.

### 2.5 AA content analysis

AA content and racemization analysis was carried out using reverse-phase high performance liquid chromatography (RP-HPLC) [91] following the methodology for unbleached samples described in Penkman et al. [92] with the following adjustment: hydrolysis was carried out using 50 μL 7M HCl (HPLC-grade, Fisher Scientific) per mg wool, previously prepared as for isotope analysis above. The following AAs were detected: aspartic acid/asparagine (Asx), glutamic acid/glutamine (Glx), serine (Ser), threonine (L-isomer only, L-Thr), histidine (L-isomer only, L-His), glycine (Gly), arginine (L-isomer only, L-Arg), alanine (Ala), tyrosine (Tyr), valine (Val), phenylalanine (Phe), leucine (Leu), and isoleucine (Ile). Experimental errors are reported in von Holstein et al. [62].

### 2.6 Statistical analysis

Statistical analysis was carried out using R [93]. Where multiple samples were tested from a single wool sample, the arithmetic mean of isotope and AA composition values was used in statistical calculations at site/settlement level. All isotope and AA data were non-parametric (univariate Shapiro-Wilk tests, P<0.001). No effective data transformations were found, so parametric statistical tests were not appropriate. Archaeological wool data are described by median and interquartile range (IQR), calculated using all data points from the site including any outliers.

The resistance of bone collagen to degradation during burial, and the consequent stability of δ^13^C and δ^15^N composition in well-preserved collagen, is well characterised [67,94], though little work has been done to confirm whether this is also true of δ^2^H values. The isotopic composition of bone collagen was used to check whether degradation has significantly altered the isotopic composition of archaeological wool samples. As collagen contains far more of the non-essential AA Gly [94], sheep bone collagen is systematically higher in δ^13^C and δ^2^H values compared to wool keratin in the same individual (2.0‰ for δ^13^C, 29‰ for δ^2^H) [53]. These offsets were used to correct collagen values in this study for comparison to keratin values.

A measure for expected isotopic range at a single site was derived from the maximal ranges of whole-year wool composition observed in modern flocks from northern Europe [31]. This data was not normal, so describing these ranges in terms of mean and standard deviation is not appropriate. Instead, flock variability was defined by the estimated standard deviation calculated via bootstrapping methods [95]. Geographic discrimination between flocks was assessed using a randomForest function, which does not assume data normality [96].

This study employed a nominal assignment framework to distinguish between local/non-local wool at each settlement tested [97]. (The use of a sheep wool isoscape was avoided because this cannot currently be modelled with any certainty, due to unsystematic baseline data availability and insufficient characterisation of the relative contributions of climatic, environmental and anthropological factors to herbivore tissue isotopic composition). Wool in archaeological textiles was identified as regionally non-local in origin if: (1) the distance of any isotope measurement from site/settlement median was more than twice the maximum 95% confidence interval for the standard deviation for that isotope in a modern sheep flock; or (2) the sample’s values were identified as outliers at site/settlement level using two robust multivariate outlier detection tests, *aq*.*plot* and *dd*.*plot* in R package *mvoutlier* [98], applied to all three isotope values.

## 3 Results

The ranges of isotopic values for each settlement are reported in Table 2. Maximum isotopic variability within samples, flocks and assemblages are compared in Table 3. Full elemental and isotopic composition results for archaeological wool are reported in S2 Table (individual textiles) and S3 Table (replicate measurements); data for archaeological bone is given in S4 Table. AA composition data are reported in S5 Table as AA concentration (pmol mg^-1^), AA % recovered and racemisation ratio (D/L). Significance testing of differences in isotopic, elemental and AA composition in both textiles and bone between settlements and regions is reported in S6 Table.

**Table 2:** Settlement median and interquartile range (maximum difference) of archaeological keratin and collagen isotope composition and C:N_atom_.

Site	Measure	δ^13^C/‰	δ^15^N/‰	δ^2^H/‰	C:N_atom_
*Keratin*					
Reykholt/IS (n = 21)	Median	-23.9	2.8	-102	3.88
	IQR	-24.1 – -23.7 (0.3)	2.4–3.9 (1.5)	-104 – -94 (11)	3.72–4.25 (0.53)
Newcastle/GB (n = 16)	Median	-24.3	6.1	-89	3.71
	IQR	-24.7 – -24.0 (0.7)	5.3–7.2 (1.9)	-90 – -87 (3)	3.63–3.76 (0.14)
Hessens/DE (n = 10)	Median	-23.3	9.7	-88	3.83
	IQR	-23.5 – -23.0 (0.5)	9.1–10.4 (1.3)	-95 – -81 (13)	3.78–3.87 (0.08)
York/GB (n = 36)	Median	-24.0	7.0	-92	3.42
	IQR	-24.2 – -23.8 (0.4)	6.2–7.6 (1.3)	-97 – -89 (8)	3.36–3.54 (0.18)
*Collagen*					
Reykholt/IS (n = 7)	Median	-21.4	3.3	-69	3.25
	IQR	-21.8 – -21.3 (0.4)	3.0–3.7 (0.7)	-72 – -69 (3)	3.23–3.31 (0.08)
Newcastle/GB (n = 29)	Median	-21.8	7.2	-59	3.26
	IQR	-21.9 – -21.5 (0.4)	5.8–8.2 (2.4)	-63 – -53 (10)	3.25–3.28 (0.03)
Hessens/DE (n = 7)	Median	-20.8	10.9	-43	3.27
	IQR	-20.9 – -20.4 (0.6)	10.2–11.7 (1.5)	-46 – -40 (5)	3.27–3.29 (0.03)
York/GB (n = 16)	Median	-22.0	6.6	-59	3.18
	IQR	-22.2 – -21.8 (0.4)	6.0–8.6 (2.6)	-65 – -55 (10)	3.17–3.20 (0.03)

**Table 3:** Maximum degree of variation in isotopic composition within a single fleece, flock, and archaeological textile (1σ).

Source of variation	δ^13^C/‰	δ^15^N/‰	δ^2^H/‰	C:N_atom_
Keratin samples, experimental error	0.2	0.4	4	/
Collagen samples, experimental error	0.2	0.4	9	/
Within single modern fleece [31]	0.5	0.7	3	0.14
Within single raw wool find (RKH 2950, n = 10)	0.1	0.3	3	0.04
Within single wool textile (YCG 4078, n = 3; *YLB 4087, n = 2)	0.6	0.5	3*	0.20
Within single modern flock (maximum basic bootstrapped 95% CI) [31]	0.7	1.7	5.8	/

### 3.1 Keratin degradation in archaeological wool samples

Modern wool exhibits C:N_atom_ values between 3.40–3.62 [31,53], higher than the theoretical values of 3.32–3.46 for the ten most abundant proteins in wool [99]. This is because C:N_atom_ also reflects the presence of the non-protein fraction of the fibre (>2% of dry mass), composed of melanins and lipids [54], which have C:N_atom_ ratios greater than 7.0. Archaeological samples in this study showed C:N_atom_ values between 3.28 and 4.54. A total of 76% of archaeological samples had C:N_atom_ values outside the modern sheep wool range, with 30% also outside the wider limits of 3.0–3.7 defined by O’Connell and Hedges [68]. Maximum range in C:N_atom_ value within a single sample was 0.36 (YCG 4078, n = 3).

C:N_atom_ value distribution was different between sites (Kruskal-Wallis test with Bonferroni correction, P<<0.001). C:N_atom_ values in York/GB assemblages (YLB and YCG; sample size in YSG was too small to test) were significantly lower than at Reykholt/IS, Hessens/DE or NBG/GB (medians 3.4 vs 3.9, 3.8 and 3.7, respectively; Mann Whitney test with Bonferroni correction, P<0.001). C:N_atom_ values were not significantly associated with any isotope ratio overall (Spearman’s rank correlation coefficient, all P>0.05), or in any individual assemblage, except at YLB/GB where a significant positive association with δ^2^H values was present (S6 Table).

**Fig 4:**
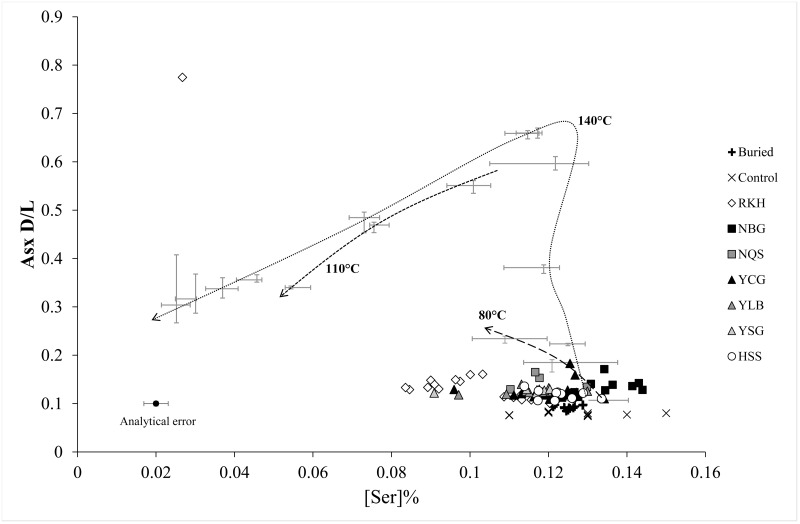
AA indicators of diagenesis in archaeological wool samples (grouped by site), compared to isothermal hydrolysis (median ± IQR per time point) and experimental burial [62]. Analytical error indicates within-sample s.d.; arrows show time series for hydrolytic experiments; error for Asx D/L is smaller than the data point.

**Fig 5:**
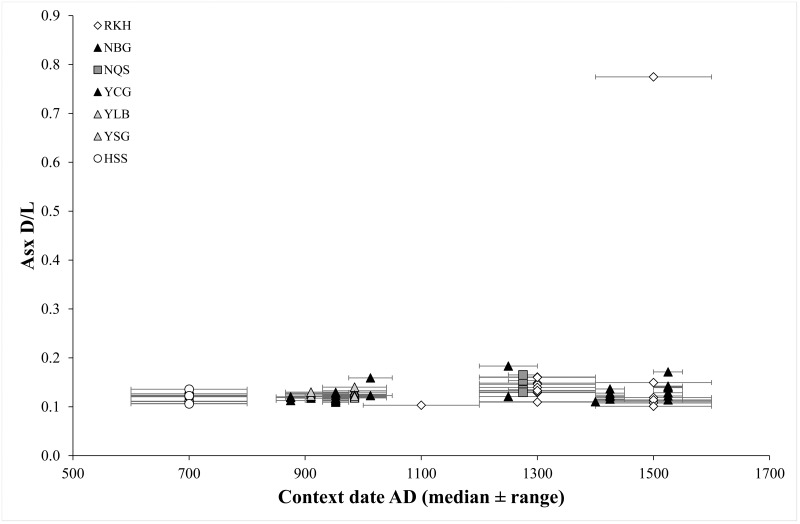
Asx D/L value against median context date for each sample. Horizontal error bars indicate total context date range. Analytical error in Asx D/L (within-sample s.d.) is smaller than the data points.

In all archaeological assemblages except Hessens/DE, the distributions of AA % contents and D/L values were significantly different from those of modern control samples (Kolmogorov-Smirnov tests, all P<0.003). AA composition of archaeological samples most resembled data from experimental burials rather than high temperature degradation [62], with low levels of racemisation, and loss of Ser (Fig 4). The highest degree and the widest range of both racemisation and hydrolysis was present in samples from Reykholt/IS, but the distribution of % AA recovered and D/L values were significantly different from those of YCG/GB and HSS/DE only (Kolmogorov-Smirnov tests with Bonferroni correction; all P<0.05). Asx D/L values were not related to sample contextual age, either within or between assemblages (Fig 5).

### 3.2 Integrity of wool isotope values as indicated by AA and elemental composition

There were very few significant correlations between AA variables (AA % composition or D/L value) and either C:N_atom_ or isotope values at any assemblage (S6 Table), and none that occurred in more than one assemblage. These correlations do not resemble the protein-specific changes observed in hydrolytically-degraded material [62], which showed a general loss of hydrophilic AAs (Asx, Gsx, Ser), relative gain of hydrophobic AAs (Phe, Ile, Leu), and decrease in δ^2^H values with increasing AA composition change. The AA variables recorded here do not detect deamidation, which has been identified proteomically in some of the same samples [63], as Asn and Gln are fully deamidated to Asp and Glu, respectively, during workup.

### 3.3 Archaeological wool keratin and bone collagen

All archaeological wool samples had higher δ^13^C values relative to modern wool samples from the same regions (archaeological range -25.3 to -22.2‰, modern range -27.6 to -25.0‰) [31]. This difference was greater than that of c. 1.5‰ expected from fossil fuel burning [58]. Differences between modern and archaeological δ^15^N value ranges were unremarkable. Absolute values of δ^2^H data are not comparable between this study and others because of differences in sample equilibration methodologies affecting absolute values and apparent H exchangeability [53,100].

**Fig 6:**
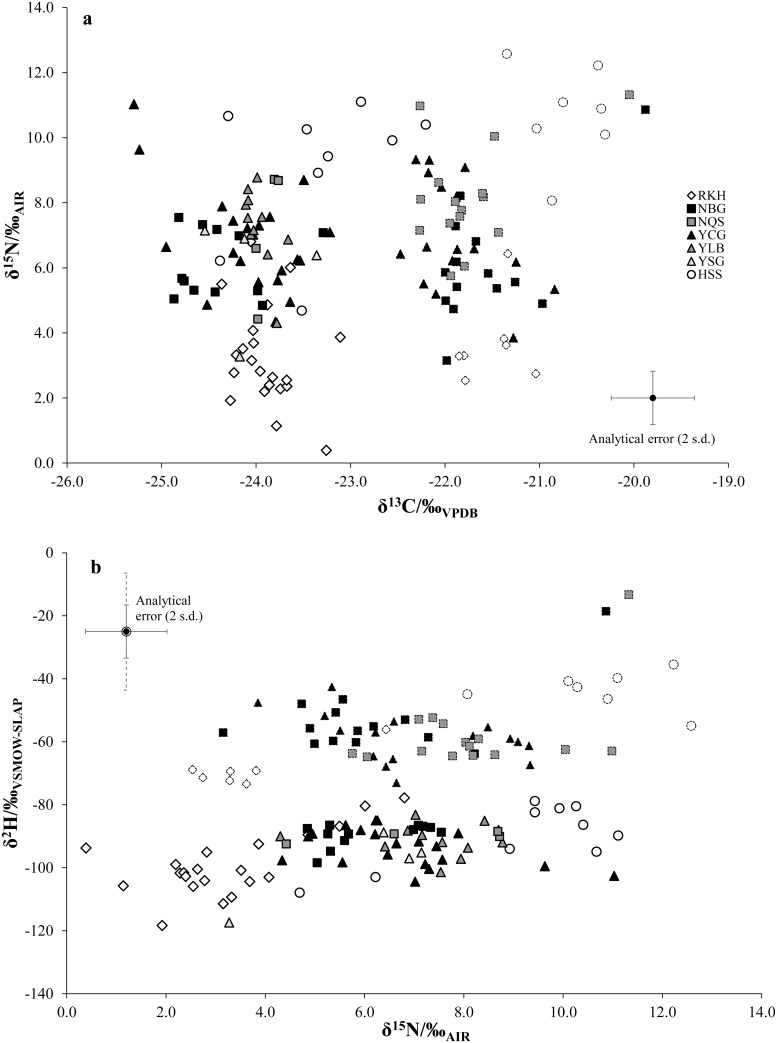
(a) δ^13^C vs δ^15^N values and (b) δ^15^N vs δ^2^H values for wool keratin samples (heavy solid outline) and bone collagen samples (light dotted outline).

**Fig 7:**
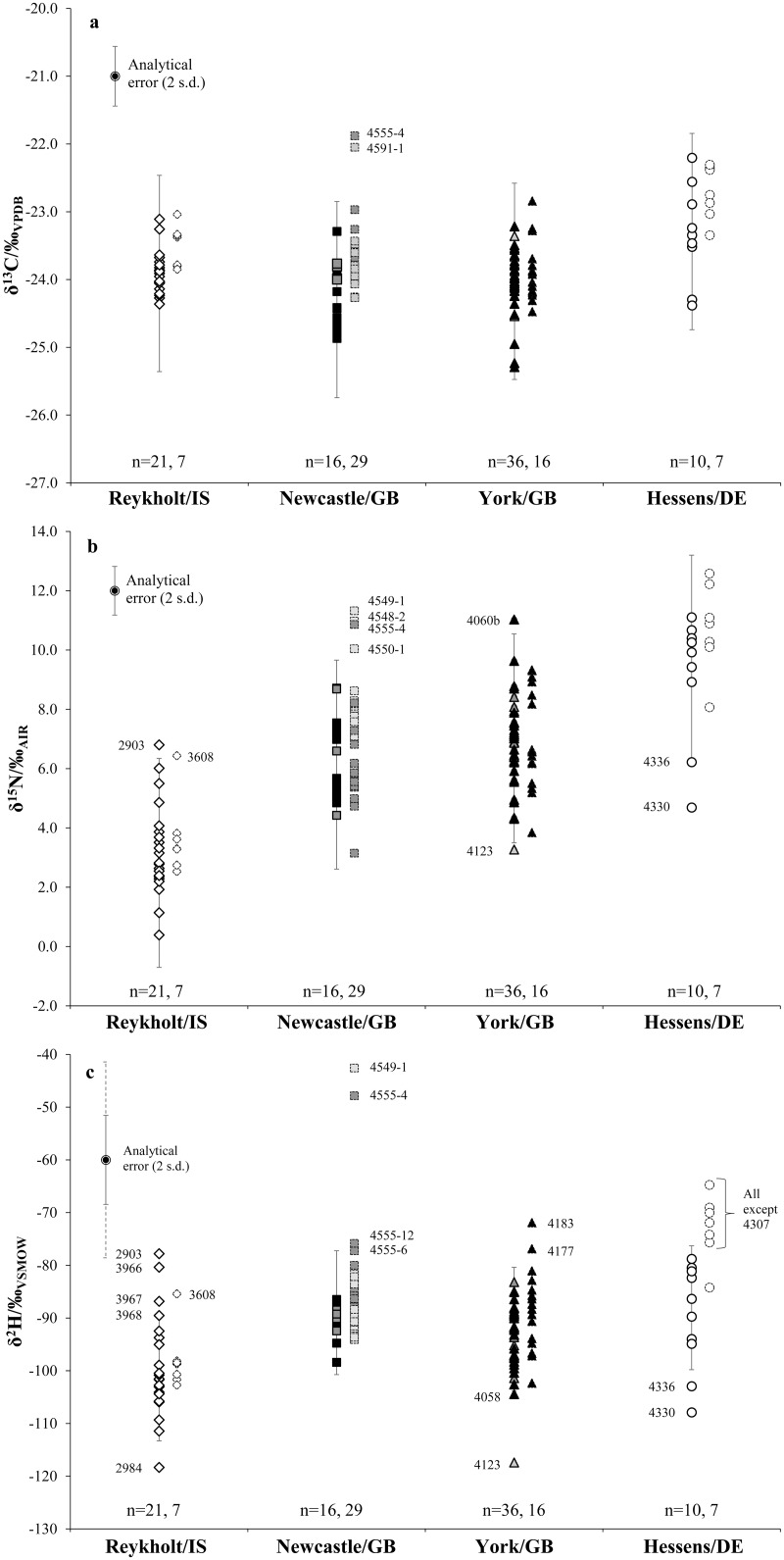
Textile keratin (left, heavy solid outlines, with error bars indicating bootstrapped maximum estimated flock range around settlement median value) and bone collagen (right, light dotted outlines) isotope values by location. (a) δ^13^C values, (b) δ^15^N values and (c) δ^2^H values. Outliers are marked by sample number. Collagen values are corrected to keratin equivalents using inter-tissue spacing data from von Holstein et al. [53].

δ^13^C, δ^15^N and δ^2^H values in both wool keratin samples and collagen bone samples clustered by region of origin (Table 4, Figs 6a–6d and 7a–7d). Material from Reykholt/IS had lower δ^15^N and δ^2^H values, and higher δ^13^C values, compared to samples from York/GB and Newcastle/GB, in parallel to the isotopic relationships identified in modern samples of sheep wool [31], sheep muscle [28] and graze plants [42,55,71]. Material from Hessens/DE was higher in both δ^13^C and δ^15^N values compared to British samples, in parallel to the salt marsh/dryland grazing offset previously identified in archaeological samples [44,50]; however modern wool showed a different relationship, with north German material showing lower δ^13^C values than and similar δ^15^N values to British material [31]. δ^2^H values were very similar between north Germany and England in archaeological wool; in modern wool, German material was similar to material from northern Britain (Penicuik) but had c. 10‰ lower δ^2^H values than wool from south-eastern Britain (Tollesbury).

**Table 4:** Textile samples with isotope compositions outlying from respective settlement median.

							In one dimension (bootstrapped estimates)			In three dimensions	
Sample type	ID	Small find no.	Site	Location	Type	Hypothesised origin	δ^13^C/‰	δ^15^N/‰	δ^2^H/‰	By *aq*.*plot*	By *dd*.*plot*
Wool keratin	2894	2001-26-30 (i)	RKH	Reykholt/IS	Yarn	typical			YES	YES	YES
	2896ave	1999-18-57	RKH	Reykholt/IS	2/2 plain twill	typical				YES	YES
	2903	1989-33-380 (f)	RKH	Reykholt/IS	Tabby	atypical		YES	YES	YES	YES
	3966	2000-6-130	RKH	Reykholt/IS	Tabby	atypical			YES	YES	YES
	3967	1989-33-380 (c)	RKH	Reykholt/IS	Tabby	atypical			YES	YES	YES
	3968	1989-33-380 (d)	RKH	Reykholt/IS	Tabby	atypical			YES	YES	YES
	4329	HE4	HSS	Hessens/DE	2/1 plain twill	atypical				YES	YES
	4330	HE21b	HSS	Hessens/DE	2/2 chevron/ diamond twill	typical		YES	YES	YES	YES
	4336	HE76c	HSS	Hessens/DE	Tabby	typical			YES	YES	YES
	4058	13584	YCG	York/GB	Staple	typical			YES		
	4060b	13382	YCG	York/GB	Yarn	typical		YES		YES	YES
	4095	10519	YCG	York/GB	Tabby	typical				YES	YES
	4123	19e	YSG	York/GB	2/2 plain twill	typical		YES	YES	YES	YES
	3949	BGT14, T5	NBG	Newcastle/GB	Tabby	typical					YES
						Totals:	0	4	9	12	13
Bone collagen	3608	/	RKH	Reykholt/IS	Humerus	/		YES	YES		
	4308	/	HSS	Hessens/DE	Mandible	/			YES		
	4309	/	HSS	Hessens/DE	Mandible	/			YES		
	4310	/	HSS	Hessens/DE	Mandible	/			YES		
	4311	/	HSS	Hessens/DE	Mandible	/			YES		
	4312	/	HSS	Hessens/DE	Mandible	/			YES		
	4313	/	HSS	Hessens/DE	Mandible	/			YES		
	4177	/	YCG	York/GB	Mandible	/			YES		
	4183	/	YCG	York/GB	Mandible	/			YES		
	4548–2	/	NQS	Newcastle/GB	Metapodial	/		YES			
	4549–1	/	NQS	Newcastle/GB	Vertebra	/	YES	YES	YES		
	4550–1	/	NQS	Newcastle/GB	Cranium	/		YES			
	4555–4	/	NBG	Newcastle/GB	Metapodial	/	YES	YES	YES		
	4555–12	/	NBG	Newcastle/GB	Metapodial	/			YES		
						Totals:	2	5	12	/	/

The offsets between median δ^13^C and δ^15^N values of bone collagen and wool keratin at each site were consistent with the metabolic offsets between these tissues in modern individual animals [53], implying that the majority of both bone and wool from each archaeological site was from animals subject to similar husbandry regimes which did not differ significantly over the life of the animal. This was also true for most δ^2^H values, except at Hessens/DE.

### 3.4 Geographic origin discrimination in archaeological samples

Within sample variation in textile samples was of the same order of magnitude as experimental error in keratin samples (Table 3). Variation in woven textiles was the same as in unprocessed wool samples at RKH; however variation within samples at York/GB was larger (S3 Table). Differences between washing methods did not increase variation in any isotope over that measured in raw staples. Within-sample variabilities were always smaller than estimated flock ranges.

For textile samples, all regions were significantly distinguished by δ^15^N values (Mann-Whitney tests with Bonferroni correction, P<0.005; S6 Table). Samples from Frisia had significantly higher δ^13^C values than those from England (P<0.05); samples from England had significantly higher δ^2^H values than those from Iceland (P<0.005). For collagen samples, all regions were significantly distinguished by δ^15^N and δ^2^H values (all P<0.005). Collagen samples from Frisia also had significantly higher δ^13^C values than those from elsewhere (P<0.05).

A randomForest function correctly classified 66% of textile samples to settlement and 77% to region. Classification of bone collagen samples to settlement was 67% correct and to region was 90% correct.

Outlier identification for textiles was most parsimonious using the bootstrapping method in one dimension (10 outlying samples), while statistical methods of outlier detection identified 12 and 13 outliers, respectively (Table 4). At each of the settlements investigated, textile samples with isotope values outlying the local range were present (Fig 7). These objects are therefore identified as of non-local provenance, with one exception (YCG/GB 4060b) where the sample was outlying in δ^15^N value only, and therefore possibly only differentiated by farming practice. At Reykholt/IS, identification of non-local material was largely consistent with textile-technical indicators of origin; at York/GB, Newcastle/GB and Hessens/DE, isotopically outlying textiles had almost all been interpreted as consistent with local manufacturing techniques, while technically atypical material was not isotopically outlying (Table 4). Surprisingly, 8 bone samples (1 at Reykholt/IS, 2 at York/GB and 5 at Newcastle/GB) were also isotopically outlying, not counting the material from Hessens/DE where wool keratin and (corrected) bone collagen ranges did not correspond well.

## 4 Discussion

### 4.1 Wool fibre integrity in archaeological samples

Analysis of AA composition of archaeological samples allowed the protein composition of these objects to be put into diagenetic context. AA composition change in archaeological samples was greater than—but comparable to—that seen in wool buried experimentally for up to 8 years; it was smaller and much less specific than the hydrolytic changes (loss of the more hydrophilic AAs, relative increase in content of the more hydrophobic AAs) observed in elevated-temperature hydrous laboratory conditions [62,63]. Archaeological samples show some hydrolytic change (particularly in the Reykholt/IS samples) but most variation is consistent with non-protein specific attack by microorganisms, in agreement with proteomic analysis of a subset of the same samples [63]. Clustering of AA variables by assemblage indicated that the primary determinant of wool fibre molecular integrity (AA composition and racemisation) was local soil environment (i.e. humidity, temperature, acidity, oxidation), but not date of context (Fig 5) or pre-burial processing (e.g. weaving, dyeing). Dating methods based on AA variables, for example Asx racemization value [101], are therefore not appropriate for buried wool samples. Overall, Reykholt/IS samples showed the highest degree of protein change, and York/GB samples the least, again in parallel to proteomic data [63], and to microscopic characterisation of fibre damage (see references in Table 1). These patterns reflect the local balance of soil characteristics which control fibre degradation (temperature, pH, microbiological activity).

According to the previously-employed measure of keratin fibre diagenesis, C:N_atom_, the majority of samples in this study were too degraded for isotopic analysis. However, AA variables indicated that elevated C:N_atom_ values were present in samples which show good protein preservation (e.g. 3950, NBG), and conversely, acceptable C:N_atom_ values were present in samples which show considerable protein change (e.g. 3962, RKH; Fig 6). C:N_atom_ reflects the composition of the whole fibre, not just the protein component, in contrast to AA data which reflects protein only. It is therefore possible that the generally high C:N_atom_ values observed in this study indicate changes in the proportion of protein to non-protein components of the fibre (i.e. relative loss of protein), or diagenesis of the non-protein moiety of the fibre. The former is more likely, as melanins are less susceptible to chemical alteration than proteins, given their heterogeneous polymeric structure and insolubility [102]. C:N_atom_ should probably not be used as a guide to the isotopic integrity of archaeological keratin samples, as it is most sensitive to changes in the proportion of protein to non-protein moieties of the fibre. This could have isotopic effects if melanins have significantly different isotopic composition to keratins. Melanins are derived from Tyr and Cys, and their presence has been shown to affect at least δ^13^C values [103]. AA-based methods are to be preferred to indicate the degree of preservation of the bulk of the fibre.

The absence of correlation between AA composition variables, C:N_atom_ and isotope values indicated that samples with outlying isotope values were not more degraded than typical samples in any assemblage. Thus, isotope composition from all archaeological samples could be interpreted as equally indicative of pre-burial values, and used to investigate provenance. The only possible exception was for wool with dense natural pigmentation. In high temperature hydrolysis experiments, a significant decrease in δ^13^C and δ^15^N (but not δ^2^H) values was observed in densely pigmented samples, without significant protein AA composition change but with deamidation [62,63]. It therefore remains possible that samples with this pigmentation might show outlying δ^13^C and δ^15^N values due to diagenetic change. The only examples of this in the present study are 4330 and 4336, both at Hessens/DE (Table 4).

### 4.2 Isotope composition of archaeological wool and bone collagen

The strong clustering of δ^13^C and δ^15^N isotope values for wool keratin and (tissue-adjusted) bone collagen samples indicated that, in accordance with wool sample AA data, keratin preservation was good, giving geographically plausible isotopic results, in line with expectations from previously published data from modern sheep muscle protein, modern vegetation samples and archaeological sheep bone [28,31,42,50,71]. Agreement between bone collagen and wool keratin δ^2^H values was also generally good, except for δ^2^H values at Hessens/DE, where (adjusted) collagen values were higher than keratin values. It is unlikely that these differences indicate that the bone and wool at this site came from animals raised in different locations, given the good agreement between tissues in δ^13^C and δ^15^N values. Instead, these variations could reflect differences in the growth periods of the two tissues. Collagen is expected to average dietary inputs over years, in contrast to keratin, which reflects inputs between shearing dates. Lower δ^2^H values in keratin suggest greater inputs from winter diet in wool than in collagen [29], which is unlikely given that wool grows faster in summer than in winter as it is under photoperiodic control [104]; winter wool is therefore unlikely to account for the bulk of textile production at a site. This result therefore at present remains unexplained.

The overall agreement between bone collagen and wool keratin isotopic composition at all settlements tested indicates the basic robustness of isotopic data derived from archaeological wool protein, and supported our hypothesis 1 (see section 2). The parallels between modern and archaeological data supported hypothesis 3. Assuming that the majority of the bone samples were of local production (as is typically assumed in isotope studies in archaeology), then so were the majority of wool samples at all sites examined.

### 4.3 Averaging of seasonal variability in wool textiles

Unlike other keratin-based archaeological materials, wool in textiles has been highly mechanically processed. Where shearing is annual, whole-year samples of wool are likely to reflect summer diet inputs more strongly than winter inputs [31]. A single yarn typically contains at least 50 fibres, so a cross-sectional sample of this is likely to return an average isotopic compositional value for the period of wool growth. At Reykholt/IS, this effect could be tested by comparison of samples 2950 (unprocessed) and 4120 (woven). Combing and weaving did not increase compositional variability over that present in the raw fleece in any isotope (S3 Table), supporting hypothesis 2. However, the magnitude of within-sample isotopic variation in finished textiles differed between assemblages, being larger in the York/GB material than in the Reykholt/IS samples. This indicates either greater seasonal variability (environmental or farming-related) in wool isotopic composition in the region supplying York/GB with wool, and/or greater farming/environmental variability in the region supplying wool to York/GB. However in no case were within-sample variabilities greater than the maximum bootstrapped estimate for within-flock variabilities. Nevertheless, the presence of a wider range of farming practices influencing wool isotopic composition in a single region has the potential to impair the geographical resolution of the technique for that region.

### 4.4 Identification of non-local textiles

The nominal assignment framework used to distinguish between local/non-local wool at each settlement in this study was based on estimates of isotopic range from annual wool samples from modern flocks in northern Europe [31]. The range criterion employed (twice the maximum bootstrapped 95% confidence interval from site median) is a deliberate overestimate of annual flock variability, in order to reduce the likelihood of Type 1 errors (incorrect identification of local material as non-local), and also to compensate for the much greater chronological range of archaeological sampling (200–600 years in this study) compared to modern samples (1–3 years). The use of a metabolically-based estimate of flock range was preferred to a statistical method (e.g. observed mean ± 2 s.d.) as it is less susceptible to sampling bias, especially as sampling deliberately included material likely to be non-local (Fig 7).

Maximum flock ranges were derived from flocks sheared annually in a single event [31]. Variability in wool shearing date may significantly increase flock isotopic variability, incorporating a different set of dietary inputs to fibre. Shearing frequency differs between farming practice regions in northern Europe (1–4 times per year) [80,105] and there is little data on frequency of shearing in the medieval period in Iceland or Frisia [106], though yearly shearing appears to have been typical in Britain [81]. It is thus possible that wool isotopic range for a site could be increased if shearing at that site was frequent and/or irregular.

The bootstrapped estimate of flock range was applied to the median point of each assemblage to identify local from non-local material. Thus, data from non-local wool was included in the calculation which established the isotopic range of local material. Potential for error from this circular reasoning was minimised by deliberately sampling many more objects considered to be local, and by comparison of the local wool median to bone collagen median isotope values.

At Reykholt/IS, isotopic results were entirely in line with the earlier interpretation of textile origin at this site, based on structural features of the finds. This contrasted with ^87^Sr/^86^Sr data from two of the samples, where exogenous (soil-derived) material obscured endogenous isotopic signals [65]. All four tabby textiles analysed here, dated by context to the 14th-16th centuries, are clearly differentiated from typical textiles at Icelandic sites in both technology and fibre type; isotopically two were outlying and the other two showed the same isotopic trends towards relatively high δ^15^N and δ^2^H. Technologically, samples 2903, 3967 and 3968 are consistent with the types of commercial production recorded in late medieval historical documents in northern mainland Europe [11]. Their δ^13^C and δ^15^N isotope values were consistent this origin, but they were more depleted in ^2^H than material from any of the other settlements tested in this study, suggesting that the wool in these samples originated further south than Britain or northern Germany, and at relatively low altitude. They probably arrived in Iceland as traded goods via late medieval trade networks [107]; earlier contexts at Reykholt/IS contain only textiles consistent with an origin in Iceland.

Isotopic results from Hessens/DE have highly significant implications for the ongoing discussion on the origin and nature of pallia fresonica [18–20]. If the term did refer to textiles manufactured in Frisia using locally produced wool, then relatively enriched δ^13^C and especially δ^15^N values, consistent with this salt-marsh grazing environment, could be a new biomarker for these textiles elsewhere. There are three samples of textile from Hessens/DE which are consistent with one of these definitions [19] (4330, 4337, 4338) but only two of these (4337 and 4338) have wool isotopic composition consistent with an origin in a salt-marsh grazing area. Samples 4330 and 4336 are instead unlikely to be from Frisia, demonstrating the integration of the small village of Hessens/DE into long-distance transfer networks in the early medieval period [108,109]. From the small sample size tested here, it is not possible to say whether these textiles were moved as goods or as belongings.

The isotopic composition of all York/GB textiles identified as atypical of English manufacture (or showing hybrid features), such as the sample of *vaðmál*-like twill (4068) and the “Coppergate sock” (3959), were nevertheless consistent with an origin in the British Isles. They could originate from a region of Britain then under strong Scandinavian influence, e.g. Viking Dublin, or the Danelaw region of England, including York itself [7,110]. The isotopic composition of wool from Denmark can be identical to that of Britain (Fig 3), and it is therefore possible that the Scandinavian-type textiles were instead made in Denmark. In contrast, sample 4123 with outlying isotopic composition could originate in northern or highland Scandinavia, by analogy with data in Fig 3 from Iceland and coastal Norway. It could have arrived in York by commercial mechanisms or as migrants’ belongings.

Of the Newcastle/GB textiles, sample 3944 (knitted cap with kermes dye in Fine-type fleece) was strongly expected to be made of Spanish or French wool [78] because the fleece type, dye and knitting itself are all unusual for the British Isles in the mid-15th century. This sample is however not isotopically outlying from the British range, suggesting that either the technique of knitting arrived in Britain earlier than previously thought, or that the garment originated from a region in Europe with a climate and environment relatively similar to that of the British Isles, thus excluding Spain and southern France.

The presence of bone collagen samples which had isotopic composition outside the local textile range (after correction for the offset between tissues) indicates that it cannot be assumed that all zooarchaeological material at a medieval site is local. This is not surprising for late medieval towns (York and Newcastle, GB) but is more so for rural sites such as Reykholt/IS and Hessens/DE. The additional uncertainty associated with the offset in isotopic composition between tissues suggests that not all of these are likely to be genuinely non-local. However it is extremely unlikely that samples 3608 (Reykholt/IS), 4549–1 (NQS/GB) and 4555–4 (NBG/GB) are local to each respective find site.

## 5 Conclusion

This study has shown that modern wool keratin, archaeological wool keratin and archaeological bone collagen δ^13^C, δ^15^N and non-exchangeable δ^2^H values show largely parallel geographic relationships in northwest Europe, and that these are comprehensible in terms of climate, grassland and farming practice differences between regions. Degradation occurring in archaeological keratin samples preserved by anoxic waterlogging did not significantly alter textile isotopic composition at any site, and did not obscure geographical origin. C:N_atom_ is not a good guide to keratin protein preservation; AA-based methods are more promising.

Using a nominal assignment framework based on the variability of wool isotopic composition within single modern flocks, it was possible to assign local/non-local origin to archaeological sheep wool samples. Wool origin could be clearly differentiated between Iceland, north-east England and Frisia, and in each region, non-local wool in textiles was identified. The degree of isotopic variability caused by environment and farming practices within a region will affect the resolution of this provenancing technique in the present and the past.

## Supporting Information

S1 Table Context and textile-technical data for all sampled textiles. (XLSX)

S2 Table δ^13^C, δ^15^N, δ^2^H and elemental composition data for all sampled textiles. For multiply-sampled objects, means are given.
(XLSX)

S3 Table δ^13^C, δ^15^N, δ^2^H and elemental composition data for all aliquots of multiply-sampled textiles. (XLSX)

S4 Table Context and textile-technical data for all sampled bone. (XLSX)

S5 Table AA concentration and racemisation results for all sampled textiles. (XLSX)

S6 Table Summary of statistical testing for: (1) associations between textile isotopic, elemental and AA variables within settlements; (2) differences in keratin and collagen isotope value between settlements and regions; (3) differences in keratin and collagen isotope distribution between settlements and regions; (4) randomForest classifications of collagen and keratin samples within regions. (XLSX)
